# Patient-reported outcomes following patellofemoral and total knee replacement: an analysis of the 6–18 months postoperative period from the National Joint Registry

**DOI:** 10.2340/17453674.2025.45115

**Published:** 2026-01-23

**Authors:** Martinique VELLA-BALDACCHINO, Alex BOTTLE, Justin COBB, Alexander D LIDDLE

**Affiliations:** 1 Msk Lab – Imperial College London, Department of Surgery & Cancer, Sir Michael Uren Hub, London; 2 School of Public Health, Imperial College London, London, UK

## Abstract

**Background and purpose:**

Patellofemoral joint replacements (PFR) and total knee replacements (TKR) are surgical treatment options for patellofemoral joint osteoarthritis. We aimed to compare patient-reported outcome measures (PROMs) for these procedures, and revision thresholds for PFR.

**Methods:**

Data from the National Joint Registry (2009–2021) was linked with the Hospital Episodes Statistics (HES) database. Data was then merged with the PROMs dataset and adjusted using inverse proportional treatment weighting methods. Primary PROMS were Oxford Knee Score (OKS) and EQ5D-3L at the 6–18-month mark with a minimal clinically important difference of 5 for OKS. Secondary outcome measures included threshold to revision, defined as the cut-off score at which an arthroplasty was revised. Differences in patient characteristics between those classed as best and worst outcomes were compared and regression analyses examined the influence of factors such as age, provider type (public or private healthcare), and comorbidities on PROMs with results stratified by sex.

**Results:**

340,449 matched records were analyzed (1,085 PFR, 339,364 TKR). The median postoperative OKS was 35 (PFR) and 38 (TKR), with a difference of –2.4. Patients with the best PFR outcomes were older (62.0 vs 57.2 years, P = 0.01). The median 6-month EQ5D-3L was 0.77 (PFR) and 0.80 (TKR). PFR had a higher revision hazard ratio (3.4, 95% confidence interval 2.7–4.4, P = 0.01), indicating a lower threshold for revision.

**Conclusion:**

Up to 18 months, in terms of OKS and EQ5D-3L, there was no significant difference between the 2 procedures. PFR had a lower threshold for revision compared with TKR. Future research should incorporate more objective measures, such as activity level, where objective differences might be identified.

Surgical treatment options for patellofemoral joint osteoarthritis include patellofemoral joint replacement (PFR) or total knee replacement (TKR) [1]. As of 1979 early designs of PFR were plagued with complications due to poor design [2]. There has been a renewed interest in PFR secondary to modern implant designs and benefits such as a quicker recovery and reduced blood loss [3]. Recent studies evaluating the performance of modern implant designs have shown similar results in patient-reported outcomes between PFR and TKR [4-7]. In contrast to the small sample sizes of randomized controlled trials, national registers have the advantage of providing data on large numbers of cases.

Previous studies have indicated lower clinical thresholds for revision of partial knee replacements such as a unicompartmental knee replacement (UKR) compared with TKR, perhaps because revision of a TKR is more complicated than converting a UKR to a TKR [8]. Similarly, revision of a PFR – another partial knee replacement – is thought to be a technically straightforward procedure, comparable to a primary TKR, whereas revision of a TKR is substantially more demanding [9,10]. However, the clinical thresholds when revising a PFR as compared with a TKR have never been assessed.

Comparative analyses between procedures used for different patterns of degenerative osteoarthritis, such as UKR vs TKR, have previously provided valuable prognostic and performance insights, and a similar approach is applied here [11]. Our study compares the performance of these procedures for degenerative osteoarthritis rather than evaluating them as interchangeable treatments for the same disease pattern. We aimed to compare patient-reported outcome measures (PROMs) after PFR and TKR, and revision thresholds for PFR.

## Methods

### Study design, data, and data linkage

**Figure 1:**
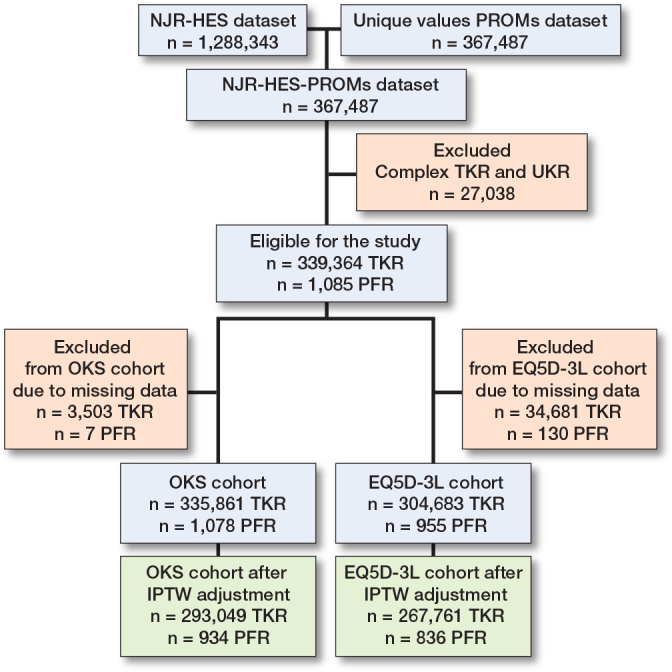
Flowchart showing the merging process between the National Joint Registry (NJR) – Hospital Episodes Statistics (HES) dataset and the Patient Reported Outcome Measures Dataset. Total knee replacements (TKRs) and patellofemoral replacements (PFRs) available for analysis are listed following removal of missing data and after adjustment of confounding with inverse proportional treatment weighting (IPTW). OKS: Oxford Knee Score.

This is a register study including PFR and primary TKR records from the National Joint Registry (NJR) from 2009 to December 2021. They were linked to the Hospital Episodes Statistics (HES) database, which is the national hospital administrative database for England, to capture hospital admissions. HES consists of all hospital admissions in the NHS and admissions for NHS patients in the private sector in England. All patients give their consent prior to being included in the NJR. The NJR has been extensively validated; completeness of primary and revision arthroplasty capture exceeds 95% with robust linkage methods [12]. This complete HES-NJR dataset was merged with the patient reported outcome measures (PROMs) dataset (Figure 1). PROMs questionnaires are electronically scanned, and patient identification numbers are traced to enable linkage with preoperative PROMs data and the HES-NJR dataset [13]. The records that were matched in both datasets were retained for analysis. A comorbidity index was calculated using a recalibration of the Charlson comorbidity index with England-calibrated weights for the International Classification of Disease and Tenth Revision (ICD-10) codes [14]. The analyses were restricted to patients older than 18 years undergoing a procedure for osteoarthritis. Observations with missing preoperative or postoperative PROMS were identified and removed. PROMs submitted between 6 and 18 months were used since the majority of improvement occurs in the first 6 months after joint replacement surgery, with only minor improvement thereafter [15]. Additionally, most records were submitted between 6 and 12 months, plateauing at 18 months. The study is reported according to STROBE guidelines.

### Confounders

Sex, age, American Society of Anesthesiologists (ASA) physical status classification system, indices of social deprivation, regional anesthesia, provider (private or national health service), operating surgeon level (consultant grade vs non-consultant grade), Charlson comorbidity index, obesity, and preoperative score (Oxford Knee Score [OKS] and EQ5D-3L) were identified. The above-mentioned confounding factors were adjusted with inverse proportional treatment weighting (IPTW) methods. Standardized differences were used to evaluate whether the treatment and control groups were comparable. A difference of less than 10% indicates good balance between groups, while a difference greater than 10% suggests that the groups were not well matched in that variable [16].

### Outcome measures

The primary outcome measures were the median postoperative OKS and EQ-5D-3L, along with the delta values representing the change from preoperative to postoperative scores for both scores calculated as postoperative value minus preoperative value (positive values reflect clinical improvement, negative values reflect deterioration). The minimal clinically important difference (MCID) in the OKS was taken as 5 [17]. The OKS consists of 12 questions, each scored on a Likert scale from 0–4, with 4 being the best outcome [18]. The OKS was then categorized using the method of Kalairajah et al. into excellent (> 41 points on the OKS), good (34 to 41), fair (27 to 33), and poor (< 27) [19]. EQ5D-3L scores were categorized using Pareto analysis, classifying patients as better, worse, the same, or non-comparable [20]. Secondary outcome measures included thresholds of revision and differences in patient characteristics between those classified as best and worst outcomes.

### Statistics

Kernel density graphs were plotted to compare the median OKS between PFR and TKR during the postoperative study period. These plots allow readers to visualize and compare the distribution of scores between the 2 groups.

A comparison of PFR patients who scored “excellent” with those who scored “poor” based on OKS categorization or those classified as “better” vs “worse” on the EQ5D-3L categorization scale was conducted using the following variables: age, preoperative score, socioeconomic status (grouped by national quintiles, with higher quintiles being the least deprived), ethnicity, and type of volume surgeon (high or low volume) [12,21].

The relationship between age at the time of surgery, type of provider, Charlson comorbidity index, and PROMs was assessed through regression analysis. PROMs for TKR and PFR were evaluated further and analyzed independently for male and female cohorts.

Continuous independent variables were assessed for a normal distribution using box plots and a t-test then followed. For categorical variables a chi-square test was performed.

Clinical threshold for revision was determined by using the last postoperative score (OKS and EQ5D-3L) obtained when a TKR or PFR was revised. A Cox regression with revision as the failure endpoint was run for both PFR and TKR. Based on the confounding factors listed earlier, the final model fit following backward selection was assessed using Akaike’s information criterion (see Supplementary data). The proportional hazards assumption was assessed using Schoenfeld residuals and time-varying coefficients included where necessary (see Supplementary data). A flexible parametric survival model was fitted using splines and degrees of freedom using the stpm2 package within Stata (StataCorp LLC, College Station, TX, USA) factoring in the time-varying coefficients, which were OKS and procedure type [22,23].

As a sensitivity analysis, the outputs of the parametric survival model were compared with the originally specified Cox model [24]. Following this, for both procedures a 5-year survival graph was plotted as a function of the OKS and EQ5D-3L.

All statistical analysis was performed in Stata (version 18; StataCorp LLC) [25]. Statistical significance was set at P < 0.05.

### Ethics, funding, and disclosures

This study was conducted using pseudo-anonymized data from a national clinical registry. The Health Research Authority guidance confirmed that ethical approval was not required. MVB is an Orthopaedic Research UK research fellow. Funding was provided by Orthopaedic Research UK to support this work. The authors have no conflicts of interest to declare. Complete disclosure of interest forms according to ICMJE are available on the article page, doi: 10.2340/17453674.2025.45115

## Results

**Figure 2:**
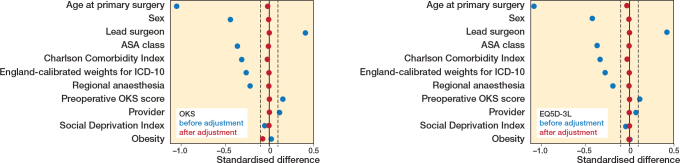
Graphical representation of inverse proportional treatment weighting – after adjustment of the Oxford Knee Score (OKS; left panel) and (EQ5D-3L; right panel).

**Figure 3:**
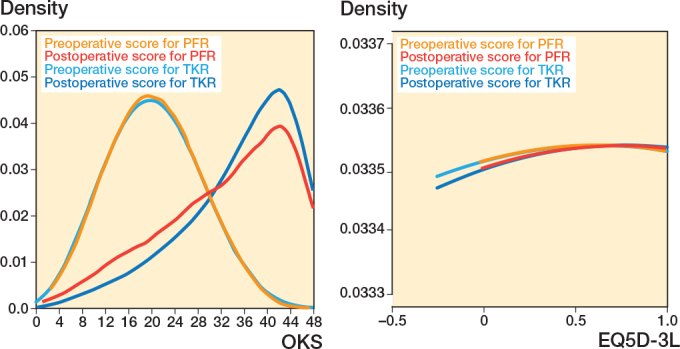
Distribution of OKS and EQ5D-3L.

There were 340,449 matched records for analysis with 1,085 PFR and 339,364 TKR (Figure 1). After exclusion of cases with missing PROM data and adjustment of confounding factors with ITPW, there were 293,049 TKR and 934 PFR with OKS and 267,761 TKR and 836 PFR with EQ5D-3L pre- and postoperative scores. The quality of confounder adjustment by IPTW is shown in Figure 2 (values can be found in Table 1 in the Appendix). Figure 2 demonstrates that all covariates were balanced within the 10% threshold after ITPW, indicating adequate comparability between the treatment and control groups. The pre- and postoperative distribution of OKS and EQ5D-3L scores within the 2 groups are presented as kernel density plots showing that PFR and TKR patients obtained similar postoperative scores (Figure 3).

### Oxford Knee Score

The median postoperative OKS for PFR was 35 (range 1–48) and 38 (range 0–48) for TKR corresponding to a median difference of –2.3 (95% confidence interval [CI] –3.0 to – 1.7, P < 0.01). Both procedures exhibited comparable score changes from preoperative to postoperative assessments, with TKR showing a median postoperative difference of 17 (range –32 to 47) and PFR demonstrating a median difference of 13 (range –24 to 40). Using categorical analysis, PFR patients were more likely to have worse OKS scores than TKR patients had, as shown in Table 2.

**Table 2:** Odds ratio (OR) of procedural success using categorization of Oxford Knee Score (OKS)

OKS categories	TKR n (%)	PFR n (%)	OR (CI)	P value	Favours
Excellent	100,982 (34)	265 (28)	0.76 (0.66–0.88	0.01	TKR
Good	93,098 (32)	254 (27)	0.79 (0.69–0.92)	0.01	TKR
Fair	48,432 (17)	161 (17)	1.10 (0.93–1.30)	0.3	None
Poor	50,537 (17)	254 (27)	1.71 (1.47–2.00)	0.01	PFR

CI: 95% confidence interval.

### Subgroup analysis

Evaluation of provider type, Charlson Comorbidity Index, social deprivation, and obesity on postoperative OKS scores showed that patients managed in the private sector demonstrated significantly higher postoperative OKS scores. Additionally, an increase in social deprivation quintile was associated with improved postoperative outcomes. Obesity was associated with lower postoperative OKS scores, with a more pronounced effect in PFR. However, the observed differences did not exceed the MCID (Table 3).

**Table 3:** Regression analysis of confounding factors influencing postoperative OKS scores

Factor	PFR	P value	TKR	P value
Age	0.19 (0.11 to 0.27)	0.01	0.14 (0.13 to 0.16)	0.01
Social deprivation quintile				
2nd	3.33 (0.52 to 6.13)	0.02	2.61 (2.22 to 3.00)	0.01
3rd	3.48 (1.13 to 5.84)	0.01	3.41 (3.10 to 3.73)	0.01
4th	5.02 (2.80 to 7.23)	0.01	4.30 (4.00 to 4.60)	0.01
5th ^a^	6.18 (3.95 to 8.41)	0.01	4.79 (4.49 to 5.08)	0.01
Provider				
private	3.58 (2.17 to 5.00)	0.01	2.00 (1.82 to 2.16)	0.01
CCI	–0.51 (–0.77 to –0.25)	0.01	–0.31 (–0.34 to –0.28)	0.01
Obese	–3.26 (–4.98 to –1.54)	0.01	–0.66 (–0.86 to –0.46)	0.01

CCI: Charlson Comorbidity Index

OKS: Oxford Knee Score

a least deprived

### Age

**Figure 4:**
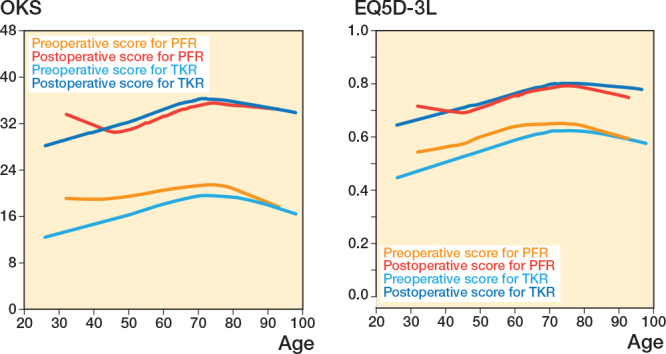
Preoperative and postoperative OKS scores (left panel) and EQ5D-3L scores (right panel) by treatment, illustrating how these scores vary depending on the age at which patients underwent surgery.

In both PFR and TKR, the postoperative OKS increased with patient age, with PFR achieving marginally better results, but not clinically significant. With every 1-year increase in age at primary PFR, there was an increase of 0.15 (CI 0.10–0.21, P = 0.01) in postoperative OKS (Figure 4) compared with 0.14 (CI 0.13–0.16, P = 0.01) for TKR.

### Sex

PFR was associated with statistically better outcomes in females compared with males. Using regression analysis with TKR as the reference category, the adjusted difference in postoperative OKS, where TKR performed better than PFR, was –1.9 (CI –2.6 to –1.1, P < 0.05) in females, compared with –4.1 (CI –5.5 to –2.6, P < 0.01) in males. This indicates that although TKR was associated with higher postoperative OKS than PFR in both sexes, the difference in OKS between TKR and PFR was smaller in females (less negative), suggesting more comparable outcomes between the 2 procedures in females. Median and range values are presented in Table 4.

**Table 4:** Median and range of postoperative Oxford Knee Score (OKS) and EQ5D-3L values by sex

Sex	OKS		EQ5D-3L	
	PFR	TKR	PFR	TKR
Male	36 (3–48)	39 (0–48)	0.77 (0.17–1)	0.81 (–0.24 to 1)
Female	35 (1–48)	37 (0–48)	0.77 (0.01–1)	0.81 (–0.24 to 1)

### Characteristics of high- and low-scoring PFR patients

Patients who had a PFR and achieved the best scores were more likely to be older compared with those who achieved the worst scores, at 62.0 (CI 60.6–63.3) compared with 57.2 (56.0–58.0); P = 0.01, respectively. They were more likely to have a higher preoperative OKS (24, CI 23–24) than those with worst scores (16, CI 15–17); P = 0.01, and more likely to have a higher preoperative EQ5D-3L, 0.70 (CI 0.68–0.72) compared with 0.51 (CI 0.49–0.54). High-volume PFR surgeons were more likely to have patients who scored “excellent” compared with low-volume surgeons OR 1.9 (CI 1.1–3.3), P = 0.03. There was no difference in terms of ethnicity.

### Threshold to revision

**Figure 5:**
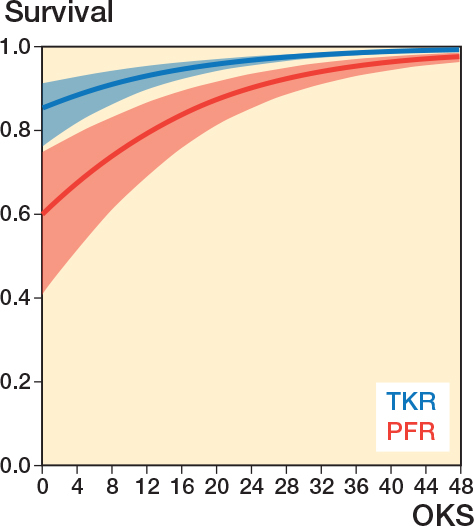
Threshold to revision at 5 years as a function of Oxford Knee Score for both PFR and TKR.

Primary PFR were more likely to be revised at a higher OKS compared with TKR and therefore had a higher revision hazard ratio: 3.2 (CI 2.4–4.1, P = 0.01 (Figure 5 and Supplementary data). Revisions were more frequent after PFR (9.2%, 79 cases) than TKR (2%, 5,575 cases).

### EQ5D-3 L

The median postoperative EQ5D-3L for PFR was 0.77 (range 0.001–1) and for TKR 0.81 (–0.24 to 1). Using a regression analysis comparing the 2 procedures, there was a difference of –0.03 (CI –0.05 to –0.00, P < 0.01). Using Pareto analysis, TKR patients were more likely to achieve better scores (Table 5).

**Table 5:** Comparison of PFR with TKR on EQ5D-3L categorical outcomes analyzed using logistic regression

EQ5D-3L categories	TKR n (%)	PFR n (%)	OR (CI)	P value	Favours
Better	208,727 (78)	548 (66)	0.49 (0.37–0.66)	0.01	TKR
Worse	22,885 (8.5)	128 (15)	2.18 (1.51–3.15)	0.01	PFR
No change	25,235 (9.4)	114 (14)	1.54 (1.02–2.33)	0.04	PFR
Mixed ch.	10,914 (4.1)	46 (5.6)	1.26 (0.66–2.42)	0.5	None

For abbreviations, see Table 2.

### Subgroup analysis

Unlike OKS, no significant statistical associations were observed between subgroup factors, including social deprivation, provider status, obesity, and the Charlson Comorbidity Index for PFR patients. In TKR, patients in the least deprived socioeconomic groups and those treated in the private sector were positively associated with higher EQ-5D-3L scores. However, higher Charlson Comorbidity Index scores and obesity were negatively associated with EQ-5D-3L (Table 6).

**Table 6:** Regression analysis of confounding factors influencing postoperative EQ5D-3L scores

Factor	PFR	P value	TKR	P value
Age	0.01 (–0.01 to 0.01)	0.08	0.01 (0.01 to 0.01)	0.01
Social deprivation quintile				
2nd	0.06 (–0.04 to 0.15)	0.3	0.03 (0.03 to 0.04)	0.01
3rd	0.04 (–0.06 to 0.14)	0.5	0.05 (0.04 to 0.05)	0.01
4th	0.05 (–0.05 to 0.15)	0.3	0.06 (0.05 to 0.06)	0.01
5th ^a^	0.09 (–0.01 to 0.19)	0.06	0.07 (0.06 to 0.07)	0.01
Provider				
private	0.03 (–0.02 to 0.09)	0.2	0.03 (0.02 to 0.03)	0.01
CCI	–0.01 (–0.02 to 0.01)	0.1	–0.01 (–0.01 to –0.01)	0.01
Obese	0.02 (–0.04 to 0.09)	0.4	–0.01 (–0.01 to –0.01)	0.01

For abbreviations, see Table 3.

### Age

T-test showed there was a significant relationship between age and EQ5D-3. When this was explored with a regression it was found that with every 1-year increase in age the EQ5D-3L for TKR increased by 0.002 (CI 0.002–0.002, P < 0.01) (see Figure 4) unlike PFR, which showed a non-significant relationship of 0.002 (CI –0.002 to 0.005, P = 0.06).

### Sex

PFR was associated with statistically better EQ-5D-3L outcomes in females compared with males. Using regression analysis with TKR as the reference category, the adjusted difference in postoperative EQ-5D-3L for PFR was –0.02 (CI –0.04 to –0.01, P < 0.01) in females, and –0.05 (CI –0.07 to –0.03, P < 0.01) in males. This indicates that, compared with TKR, PFR in females results in a smaller reduction and therefore a smaller difference in EQ-5D-3L outcomes between the 2 procedures than observed in males. Median and range values are presented in Table 3.

### Characteristics of high- and low-scoring PFR patients

Similar to OKS, patients who had a PFR and achieved better scores were more likely to be the least deprived (P = 0.01). However, there was no difference in ethnicity or if the surgeon was classified as a high- or low-volume PFR surgeon (see Supplementary data).

### Threshold to revision

**Figure 6:**
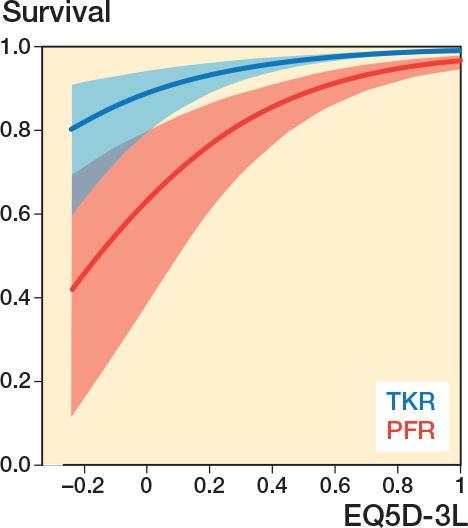
Threshold to revision at 5 years as a function of EQ5D-3L for both PFR and TKR.

By adjusting for age at primary surgery (Figure 6), PFRs were more likely to be revised at a higher EQ5D-3L compared with TKR and therefore have a higher hazard ratio 3.40 (CI 2.66-4.35).

## Discussion

Our study is the largest PROMS registry study conducted on PFR. We aimed to compare the PFR and TKR using OKS, EQ5D-3L, and revision thresholds. We found that the postoperative OKS and EQ5D-3L did not show a clinically significant difference between PFR and TKR between 6 and 18 months. Despite the higher revision rate of PFR compared with TKR, this study demonstrates that patients undergoing a PFR can expect outcomes similar to those of patients undergoing TKR.

In terms of quality of life, there was no difference between PFR and TKR. This differed from the results of level 1 studies, favouring PFR at 6 months, but did not differ after that time [5,6]. For both procedures, those who scored “excellent” in the OKS were more likely to be older, with a mean age of 62, whereas those who scored poorly were more likely to be younger, with a mean age of 57. The clinical threshold for revision of a PFR was significantly lower than that of a TKR. Based on these findings, patients undergoing a PFR, who had otherwise similar characteristics and symptom severity as those undergoing a TKR, were more likely to be revised

### Limitations

One of the main limitations of this study is that it used only 2 study metrics, which were both patient-reported outcomes, with the OKS well known for its ceiling effects [26]. In arthroplasty registries, imaging data are not linked to individual patient records, limiting their value in evaluating outcomes. Consequently, it is uncertain what patterns of osteoarthritis patients undergoing both PFR and TKR had. However, Scott et al. demonstrated that the pattern and severity of osteoarthritis do not consistently influence outcomes [27]. This finding aligns with previous high-impact studies comparing outcomes across different osteoarthritis distributions [11].

### Conclusion

Within the first 18 months, with a MCID of 5, there was no significant difference in the OKS and EQ5D-3L between PFR and TKR. Surgeons were more willing to revise PFR when both PFR and TKR had the same PROM score. These findings indicate that patients undergoing PFR for isolated patellofemoral osteoarthritis can expect results similar to those undergoing TKR for primary knee osteoarthritis.

## Supplementary Material



## Supplementary data

Supplementary Tables 1–10 and Supplementary Figures 1–5 are available as supplementary data on the article page, doi: 10.2340/17453674.2025.45115

## Notes

MVB Writing, review and editing, methodology, conceptualization, data analysis. AB, JC, ADL: writing, review, editing, supervision.

Handling co-editor: Per Henrik Randsborg

*Acta* thanks Elizabeth A Arendt and Mika Niemeläinen for help with peer review of this manuscript.

## Appendix

**Table 1a:** Balance of covariates before and after inverse proportional treatment weighting (IPTW) for Oxford Knee Score (OKS)

	Before adjustment - OKS					After adjustment - OKS				
	TKR (n = 335,861)		PFR (n = 1,078)			TKR (n = 293,049)		PFR (n = 934)		
	Mean weight	n (%)	Mean weight	n (%)	SMD	Mean weight	n (%)	Mean weight	n (%)	SMD
Preoperative OKS	19.2		20.4		0.16	20.4		20.4		0.00
Age	70.4		59.9		-1.05	60.0		60.0		-0.02
Sex	0.43		0.23		-0.43	0.23		0.23		-0.01
Female		190,435 (57)		828 (77)			166,237 (57)		719 (77)	
Male		145,421 (43)		250 (23)			126,812 (43)		215 (23)	
missing		5 (0)		0 (						
ASA	2.08		1.90		-0.36	1.90		1.90		-0.01
ASA class 1		28,217 (8)		205 (19)			25,142 (9)		182 (19)	
ASA class2		250,911 (75)		782 (73)			220,318 (75)		670 (72)	
ASA class3		56,733 (17)		91 (8.4)			47,589 (16)		82 (1)	-0.01
Social deprivation index (in quintiles)	3.39		3.31		-0.05	3.31		3.31		-0.01
1st - most deprived		43,221 (13)		165 (15)			41,503 (14)		159 (17)	
2nd		30,954 (9.2)		88 (8.2)			29,629 (10)		88 (9.4)	
3rd		74,335 (22)		216(20)			71,379 (24)		212 (23)	
4th		80,447 (24)		261 (24)			77,332 (26)		254 (27)	
5th - least deprived		76,288 (23)		230 (21)			73,206 (25)		221 (24)	
missing		30,616 (9.1)		118 (10)						
Regional anaesthesia	0.77		0.68		-0.21	0.68		0.68		-0.00
Yes		76,360 (23)		348 (32)			65,981 (23)		300 (32)	
No		259,501 (77)		730 (67)			227,068 (77)		634 (68)	
Provider	0.28		0.33		0.12	0.34		0.34		0.00
NHS		244,624 (73)		729 (68)			210,324 (72)		619 (66)	
Private		91,192 (27)		348 (32)			82,725 (28)		315 (34)	
missing		45 (0)		1 (0)						
Lead surgeon	0.81		0.94		0.41	0.94		0.94		0.00
Consultant		271,305 (81)		1,013 (94)			237,870 (81)		882 (94)	
Non-consultant		64,556 (19)		65 (6.0)			55,179 (19)		52 (5.6)	
CCI	0.64		0.41		-0.32	0.42		0.41		-0.02
None		175,066 (52)		719 (67)			155,133 (53)		630 (67)	
Mild		115,385 (34)		286 (27)			99,191 (34)		241 (26)	
Moderate		33,248 (10)		55 (5.1)			28,486 (9.7)		48 (5.1)	
Severe		12,162 (3.6)		18 (1.7)			10,239 (3.5)		15 (1.6)	
Recalibrated CCI **^a^**	2.44		1.55		-0.27	1.56		1.56		-0.00
Obesity	0.19		0.20		0.02	0.23		0.20		-0.10

ASA = American Society of Anesthesiologists Physical Status classification

CCI = Charlson comorbidity index.

SMD = standardised mean difference.

a Recalibration of CCI with England–calibrated weights.

**Table 1b:** Balance of covariates before and after inverse proportional treatment weighting (IPTW) for EQ5D-3L

	Before adjustment - EQ5D-3L					After adjustment - EQ5D-3L				
	TKR (n = 304,683)		PFR (n = 955)			TKR (n = 267,761)		PFR (n = 836)		
	Mean weight	n (%)	Mean weight	n (%)	SMD	Mean weight	n (%)	Mean weight	n (%)	SMD
Preoperative EQ5D-3L	0.61		0.63		0.07	0.63		0.63		0.00
Age	70.3		59.6		-1.08	60.0		60.0		-0.02
Sex	0.44		0.24		-0.42	0.24		0.24		-0.01
Female		170,344 (56)		723 (76)			149,936 (56)		634 (76)	
Male		133,334 (44)		232 (24)			117,825 (44)		202 (24)	
ASA	2.08		1.89		-0.37	1.89		1.89		-0.01
ASA class 1		25,831 (8.5)		185 (19)			23,195 (8.7)		166 (20)	
ASA class 2		226,974 (75)		691 (72)			201,235 (75)		596 (71)	
ASA class 3		50,878 (17)		79 (8.3)			43,331 (16)		74 (8.9)	
Social deprivation index (in quintiles)	3.38		3.32		-0.05	3.33		3.32		-0.00
1st - most deprived		38,817 (13)		143 (15)			37,599 (14)		142 (17)	
2nd		27,900 (9.2)		79 (8)			26,978 (10)		79 (9,5)	
3rd		67,144 (22)		192 (20)			65,105 (24)		189 (23)	
4th		73,007 (24)		228 (24)			70,908 (26)		225 (27)	
5th - least deprived)		69,186 (23)		205 (21)			67,171 (25)		201 (24)	
missing		27,629 (9.1)		108 (11)						
Regional anaesthesia	0.78		0.69		-0.20	0.69		0.69		-0.00
Yes		235,114 (77)		659 (69)			207,958 (78)		579 (69)	
No		68,569 (23)		296 (31)			59,803 (22)		257 (31)	
Provider	0.28		0.34		0.12	0.34		0.34		0.00
NHS		220,796 (72)		645 (68)			191,733 (72)		553 (66)	
Private		82,844 (27)		309 (32)			76,028 (28)		283 (34)	
Lead surgeon	0.81		0.95		0.42	0.95		0.95		0.00
Consultant		245,518 (81)		900 (94)			217,148 (81)		792 (95)	
Non-consultant		58,165 (19)		55 (5.8)			50,613 (19)		44 (5.3)	
CCI	0.64		0.40		-0.34	0.42		0.40		-0.03
None		158,881 (52)		646 (68)			142,189 (53)		570 (68)	
Mild		104,144 (34)		247 (26)			90,483 (34)		212 (25)	
Moderate		29,804 (10)		49 (5.1)			25,857 (10)		43 (5,1)	
Severe		10,854 (3.6)		13 (1.4)			9,232 (3.5)		11 (1.3)	
Recalibrated CCI a	2.42		1.52		-0.30	1.52		1.52		-0.00

For abbreviations, see Table 1a.
