# Supplementary material for: Patient-reported outcomes following patellofemoral and total knee replacement: an analysis of the 6–18 months postoperative period from the National Joint Registry
Source: Acta Orthop. 2026 Jan 23;97:42–9. doi: 10.2340/17453674.2025.45115 (PMC12829338; doi:10.2340/17453674.2025.45115)
Supplement: Supplementary file 1 [file ActaO-97-45115-s1.pdf]

Supplementary data

Table 1. Summary of statistical tests on OKS categories, excellent and poor

	Test type	P value
Age	T-test	0.01
Pre-operative OKS	T-Test	0.01
Pre-operative EQ5D-3L	T-Test	0.01
Ethnicity	Chi squared	0.5
High volume: Low volume surgeon	Chi squared	0.03

Table 2. Model building for clinical threshold to revision for OKS. The table shows backward selection starting with the model including all potential predictors. Variables with the largest p-value above the significance level are eliminated one at a time from the model. The model is then refitted and the process repeated

	**Model 1 Hazard ratio (CI)**	P	**Model 2 Hazard ratio (CI)** Removal of obesity	P	**Model 3 Hazard ratio (CI)** Removal of sex	P	**Model 4 Hazard ratio (CI)** Removal of provider	P	**Model 5 Hazard ratio (CI)** Removal of social deprivation	P
6-month postop. OKS	0.94 (0.92–0.95)	0.01	0.94 (0.92–0.95)	0.01	0.94 (0.92–0.95)	0.01	0.94 (0.92–0.95)	0.01	0.94 (0.93–0.96)	0.01
Procedure										
PFR	3.46 (2.70–4.45)	0.01	3.49 (2.71–4.50)	0.01	3.50 (2.72–4.51)	0.01	3.40 (2.66–4.36)	0.01	3.40 (2.66–4.35)	0.01
Sex										
Female	1 [Reference]									

Male	1.44 (0.97–2.15)	0.10	1.41 (0.95–2.11)	0.09						
Provider										
NHS	1 [Reference]									
Private	1.45 (1.00–2.16)	0.06	1.46 (0.97–2.19)	0.07	1.45 (0.96 – 2.17)	0.08				
Age	0.99 (0.97–1.00)	0.10	0.99 (0.97–1.00)	0.11	0.99 (0.97–1.00)	0.14	0.99 (0.97–1.00)	0.16	0.99 (0.98–1.00)	0.20
Social deprivation										
2nd	1.14 (0.61–2.15)	0.68	1.15 (0.61–2.17)	0.66	1.17 (0.62–2.21)	0.63	1.18 (0.63–2.25)	0.60		
3rd	1.09 (0.65–1.82)	0.73	1.10 (0.66–1.85)	0.71	1.13 (0.68–1.90)	0.64	1.19 (0.71–1.99)	0.51		
4th	1.34 (0.80–2.26)	0.33	1.32 (0.77–2.24)	0.31	1.30 (0.76–2.21)	0.33	1.33 (0.78–2.26)	0.30		
5th	0.96 (0.53–1.74)	0.90	0.97 (0.54–1.75)	0.91	0.97 (0.53–1.75)		1.10 (0.56–1.82)	0.97		
Obesity										
Not obese	1 [Reference]									
Obese	0.85 (0.52–1.36)	0.52								

Table 3. Final model with age transformation_transformation of the variable age (quadratic) in order to improve the model fit as it is deemed to be a clinically important variable

	**Model 1 Hazard ratio (CI)**	P value
6-month postop. OKS	0.94 (0.93-0.95)	0.01
Procedure type		
PFR	3.60 (2.81–4.61)	0.01
Age##age	1.00 (1.00–1.00)	0.01

Table 4. Exploring time varying coefficients with finalised Cox regression model. The table lists variables from a Cox regression model with P values less than 0.05 indicating time varying coefficients

	**Cox regression model Hazard ratio**	**P value**	**Time varying effects hazards ratio**	**P value**
6-month postop. OKS	0.94 (0.93–0.95)	0.01	1.04 (1.02–1.06)	0.01
Procedure type				
PFR	3.60 (2.81–4.61)	0.01	1.73 (1.25–2.40)	0.01
Age##age	1.00 (1.00–1.00)	0.01	1.00 (1.00–1.00)	1.00

Table 5. Comparison of cox regression model with flexible parametric survival model. It can be seen that the flexible parametric survival model with splines and degrees of freedom are conceptually very similar to the Cox models (and provide similar estimates), but offer advantages over Cox models if hazard ratios are not the only quantity one wants to estimate

	**Cox regression model Hazard ratio**	**Flexible parametric survival model using splines and degrees of freedom (Stpm2) Hazard ratio**
6-month postop. OKS	0.90 (0.88–0.92)	0.93 (0.92–0.94)
Procedure type		
PFR	2.01 (1.31–3.10)	3.15 (2.44–4.07)
Age##age	1.00 (1.00–1.00)	1.00 (1.00–1.00)
**AIC**	**1278.37**	**785.88**

Table 6. Model building for clinical threshold to revision for EQ5D-3L. The table shows backward selection starting with the model including all potential predictors. Variables with the largest p-value above the significance level are eliminated one at a time from the model. The model is then refitted and the process repeated

	**Model 1 Hazard ratio (CI)**	P	**Model 2 Hazard ratio (CI)** Removal of obesity	P	**Model 3 Hazard ratio (CI)** Removal of provider	P	**Model 4 Hazard ratio (CI)** Removal of social deprivation	P	**Model 5 Hazard ratio (CI)** Removal of sex	P
Postop. EQ5D-3L	0.11 (0.05 – 0.24)	0.01	0.11 (0.05–0.24)	0.01	0.11 (0.05–0.25)	0.01	0.11 (0.05–0.24)	0.01	0.12 (0.05–0.24)	0.00
Procedure type										
PFR	3.90 (3.01 – 5.02)	0.01	3.89 (3.02–5.01)	0.01	3.84 (3.00 – 4.91)	0.01	3.84 (3.00–4.92)	0.01	3.83 (3.00–4.92)	0.00
Sex										
Female	1 [Reference]									
Male	1.48 (0.99–2.21)	0.06	1.48 (1.00–2.20)	0.06	1.47 (0.99–2.20)	0.06	1.48 (1.00–2.20)	0.05		
Provider										
NHS	1 [Reference]									
Private	1.21 (0.79–1.84)	0.40	1.21 (0.79–1.85)	0.38						
Age	0.98 (0.96–1.00)	0.04	0.98 (0.96–1.00)	0.04	0.98 (0.96–1.00)	0.04	0.98 (0.97–1.00)	0.05	0.98	0.08
Social deprivation										
2nd	1.15 (0.58–2.29)	0.69	1.15 (0.58–2.28)	0.67	1.16 (0.58–2.30)	0.67				
3rd	1.17 (0.68–2.00)	0.59	1.17 (0.68–2.00)	0.58	1.19 (0.70–2.04)	0.52				
4th	1.18 (0.67–2.08)	0.56	1.18 (0.67–2.08)	0.57	1.20 (0.69–2.11)	0.52				
5th	0.92 (0.50–1.71)	0.80	0.92 (0.50–1.71)	0.80	0.94 (0.51–1.73)	0.85				
Obesity										
Not obese	1 [Reference]									

Obese	1.00 (0.62–1.64)	0.99								

Table 7. Final model with age transformation of the variable age (quadratic) in order to improve the model fit as it is deemed to be a clinically important variable

	**Model 1 Hazard ratio (confidence interval)**	P value
Postop. EQ5D-3L	0.11 (0.05–0.23)	0.01
Procedure type		
PFR	3.95 (3.08–5.07)	0.01
Age##age	1.00 (1.00–1.00)	0.16

Table 8. Exploring time varying coefficients with finalised cox model. The table lists variables from a Cox Regression model with p values less than 0.05 indicating time varying coefficients

	**Cox regression model Hazard ratio**	**P value**	**Time varying effects hazards ratio**	**P value**
Post-op EQ5D-3L	0.11 (0.05–0.23)	0.01	4.44 (2.41–8.18)	0.01
Procedure type				
PFR	3.95 (3.08–5.07)	0.01	1.70 (1.21–2.37)	0.01
Age##age	1.00 (1.00–1.00)	0.16	1.00 (1.00–1.00)	0.05

Table 9. Comparison of cox regression model with flexible parametric survival model

	**Cox regression model Hazard ratio**	**Flexible parametric survival model using splines and degrees of freedom (Stpm2) Hazard ratio**
Postop. EQ5D-3L	0.11 (0.05–0.23)	0.08 (0.40–0.15)
Procedure type		
PFR	3.95 (3.08–5.07))	3.50 (2.72–4.53)
Age##age	1 (1–1)	1 (1–1)
**AIC**	**1204**	**762**

Table 10. Summary of statistical tests on EQ5D-3L better and worse categories using Pareto analysis

	Test type	P value
Age	T-test	0.04
Social deprivation	Chi squared	0.01
Ethnicity	Chi squared	0.24
High volume: Low volume surgeon	Chi squared	0.23
Preoperative EQ5D-3L	T-test	0.01

Figures

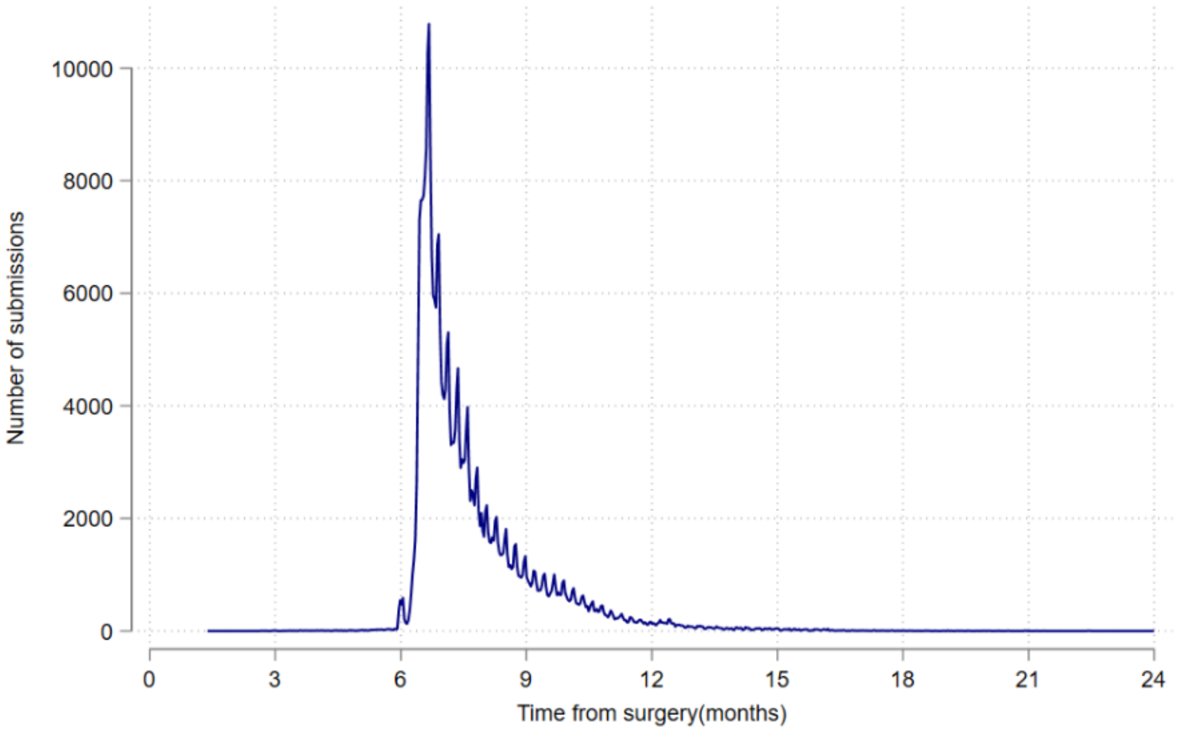

Number of submissions for OKS over time

Figure 1a. Distribution of OKS submissions over time, with peak between 6 and 12 months post surgery.

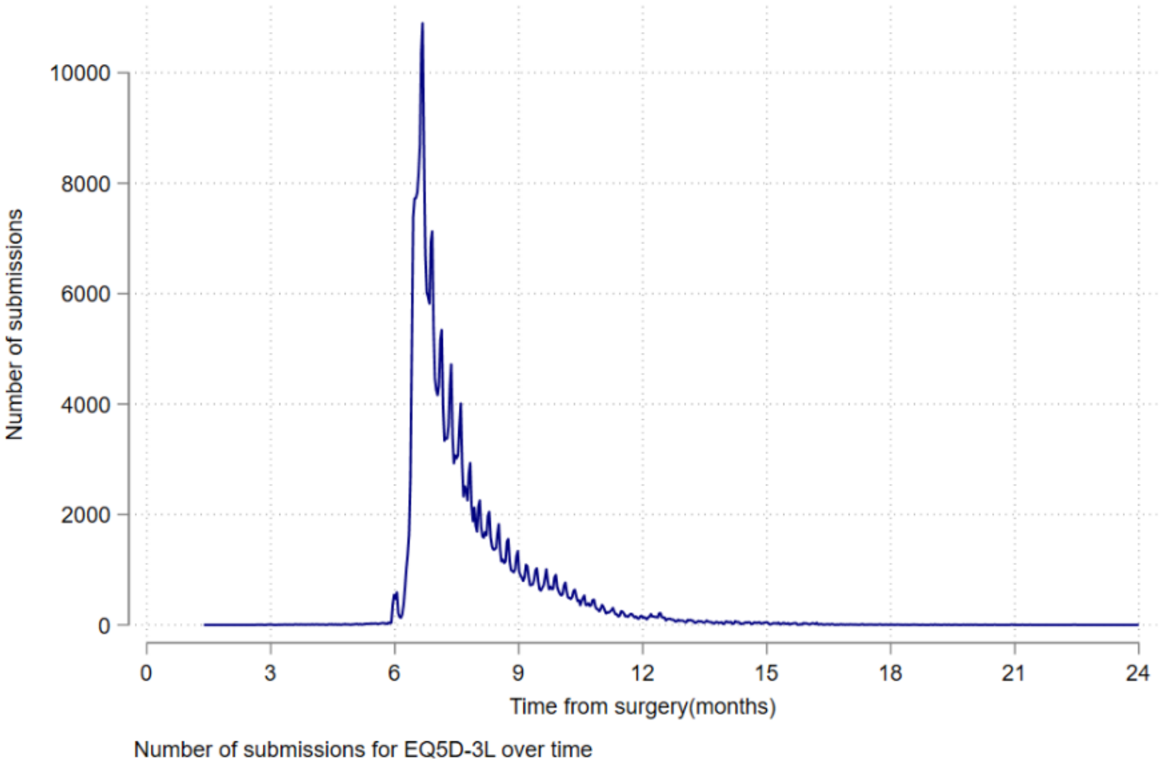

Figure 1b. Distribution of EQ5D-3L submissions over time, with peak between 6 and 12 months post surgery.

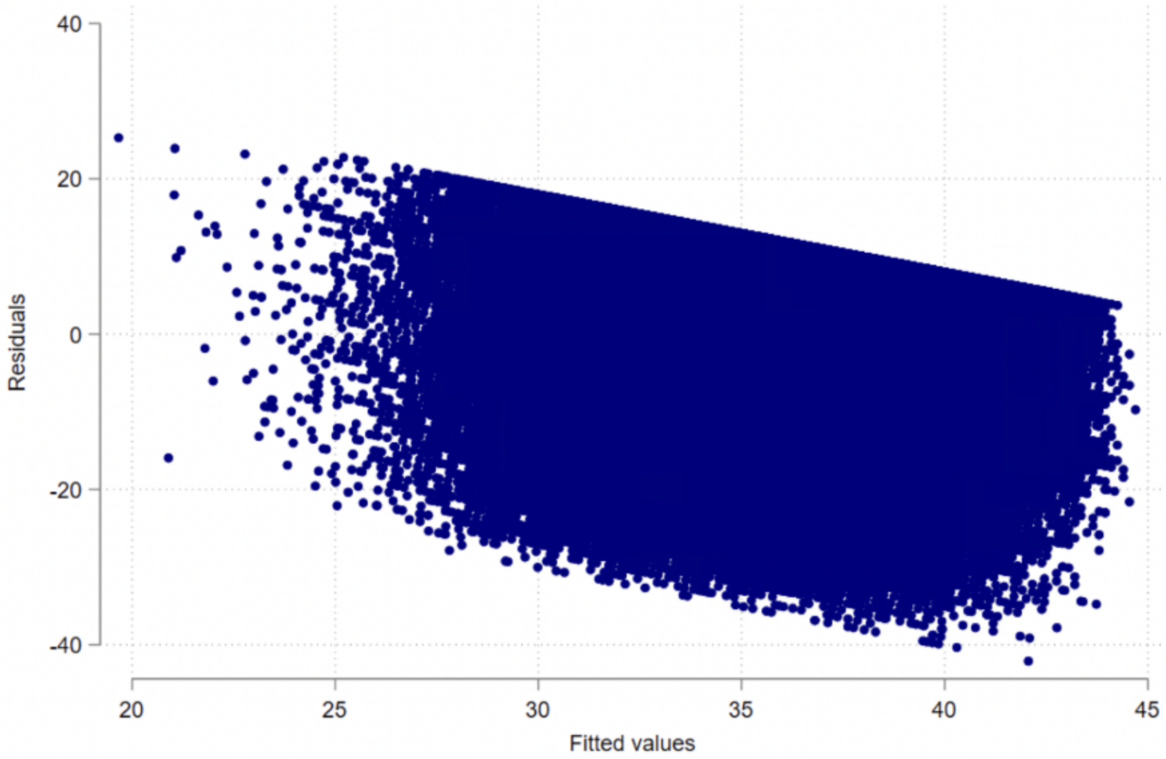

Figure 2. Scatterplot of OKS shows residuals on the y-axis and fitted values on the x-axis. It helps detect non-linearity, unequal error variances, and outliers.

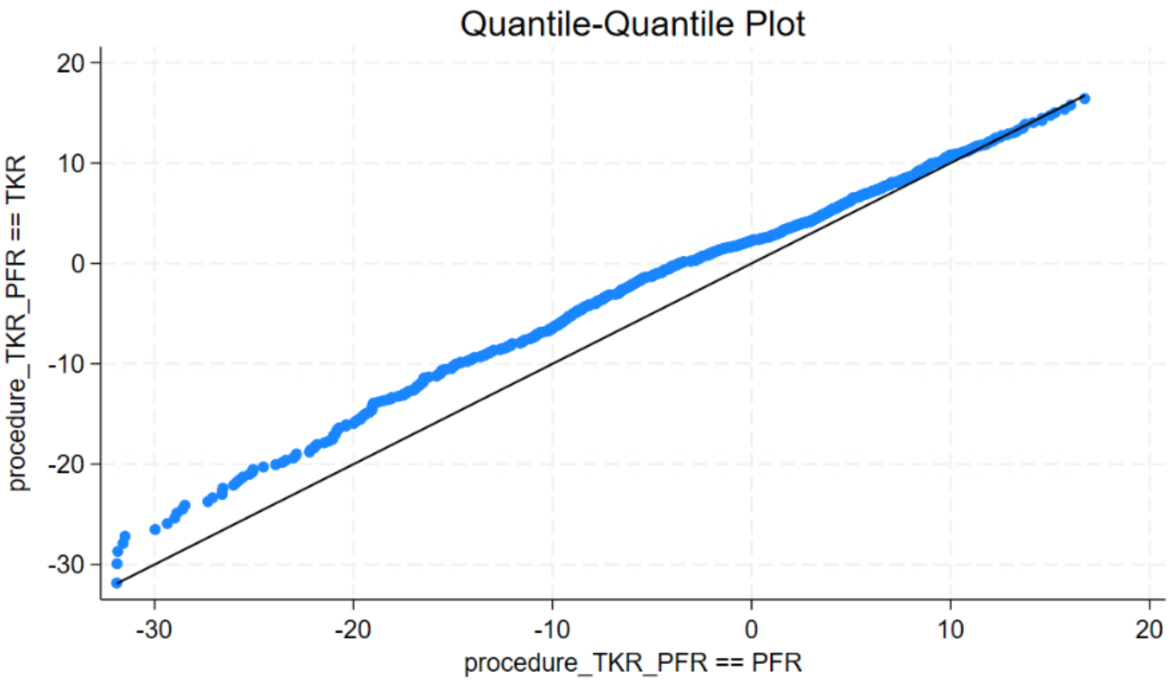

Figure 3. Quantile-quantile plot for OKS residuals the distribution of residuals to a normal distribution. Points should lie approximately along the reference line if residuals are normally distributed. Deviations from the line indicate departures from normality.

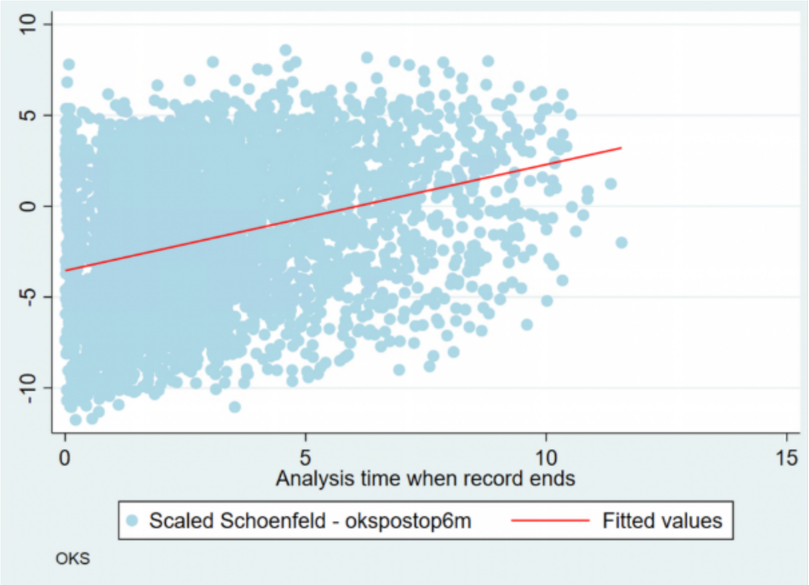

Figures 4a. Schoenfeld residuals for testing proportional hazards assumption for covariate type of procedure.

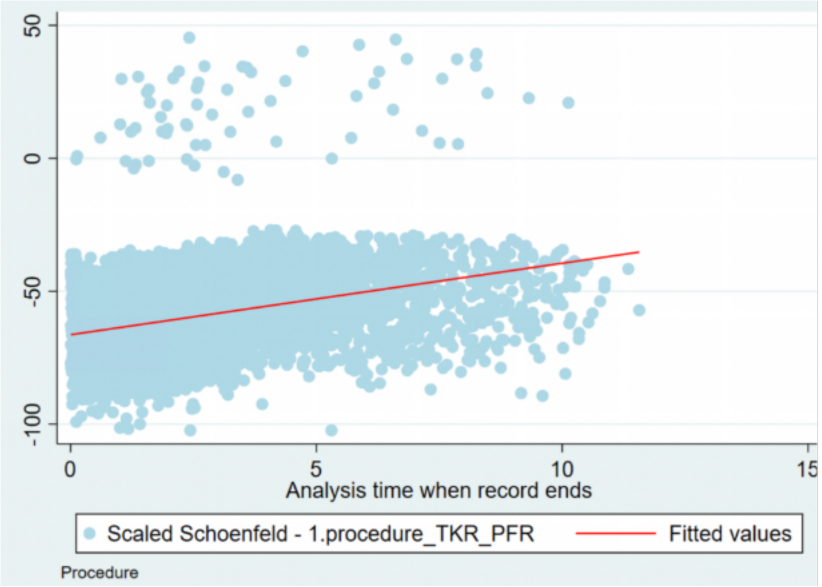

Figures 4b. Schoenfeld residuals for testing proportional hazards assumption for covariate quadratic age.

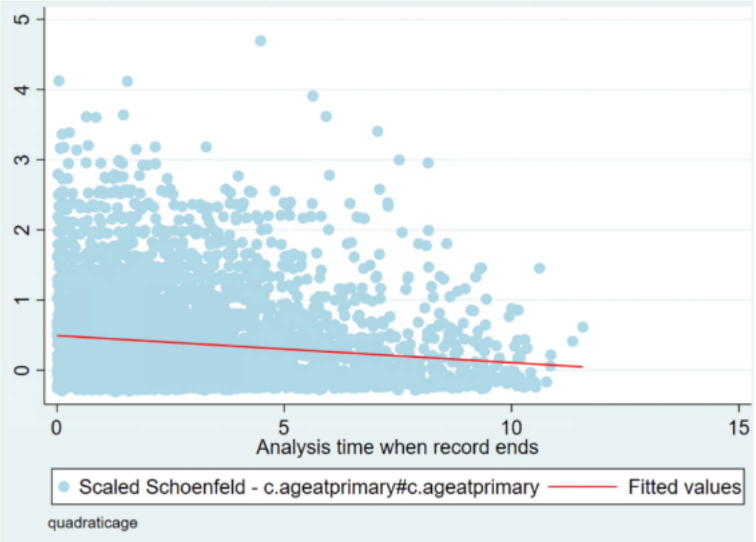

Figures 4c. The Schoenfeld residuals are calculated for each variable to see if each variable independently satisfies the assumptions of the Cox model. These are presented graphically, whereby if the covariate satisfies proportional hazards, the line should be flat centred around zero.

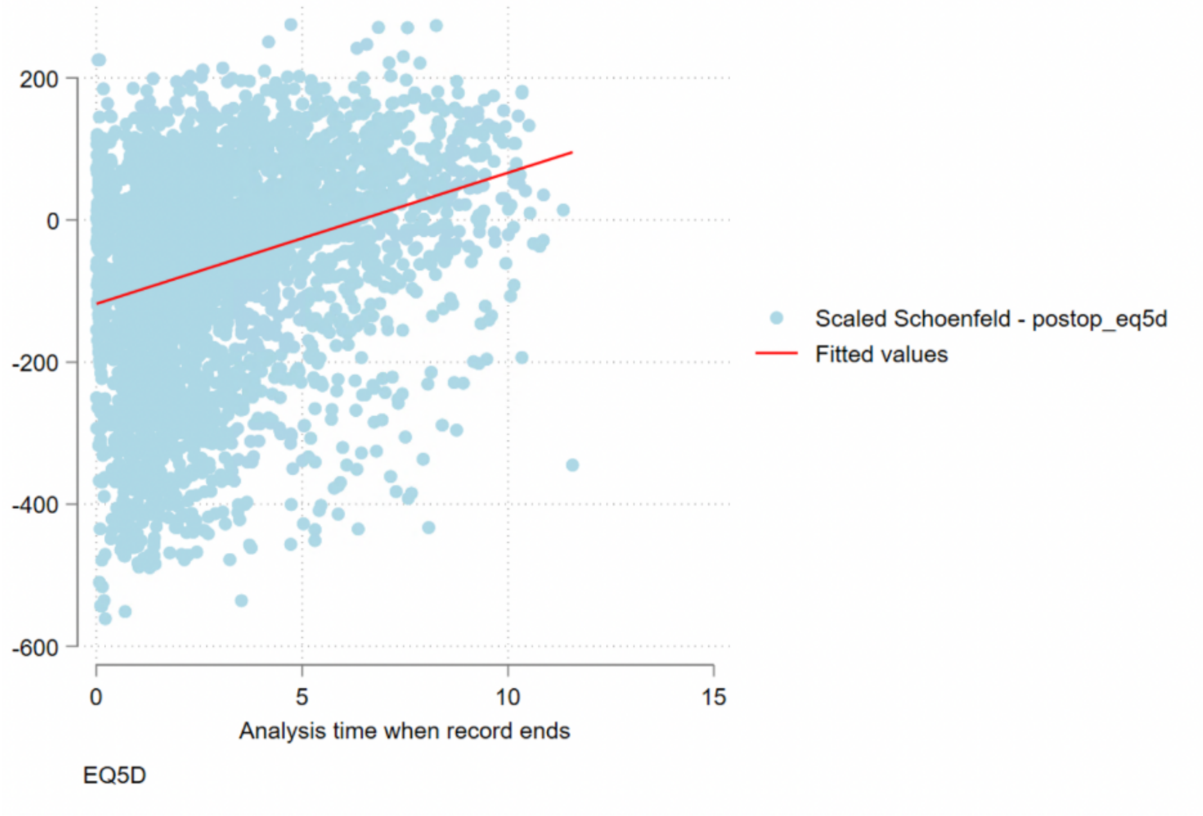

Figures 5a. Schoenfeld residuals for testing proportional hazards assumption for covariate EQ5D-3L.

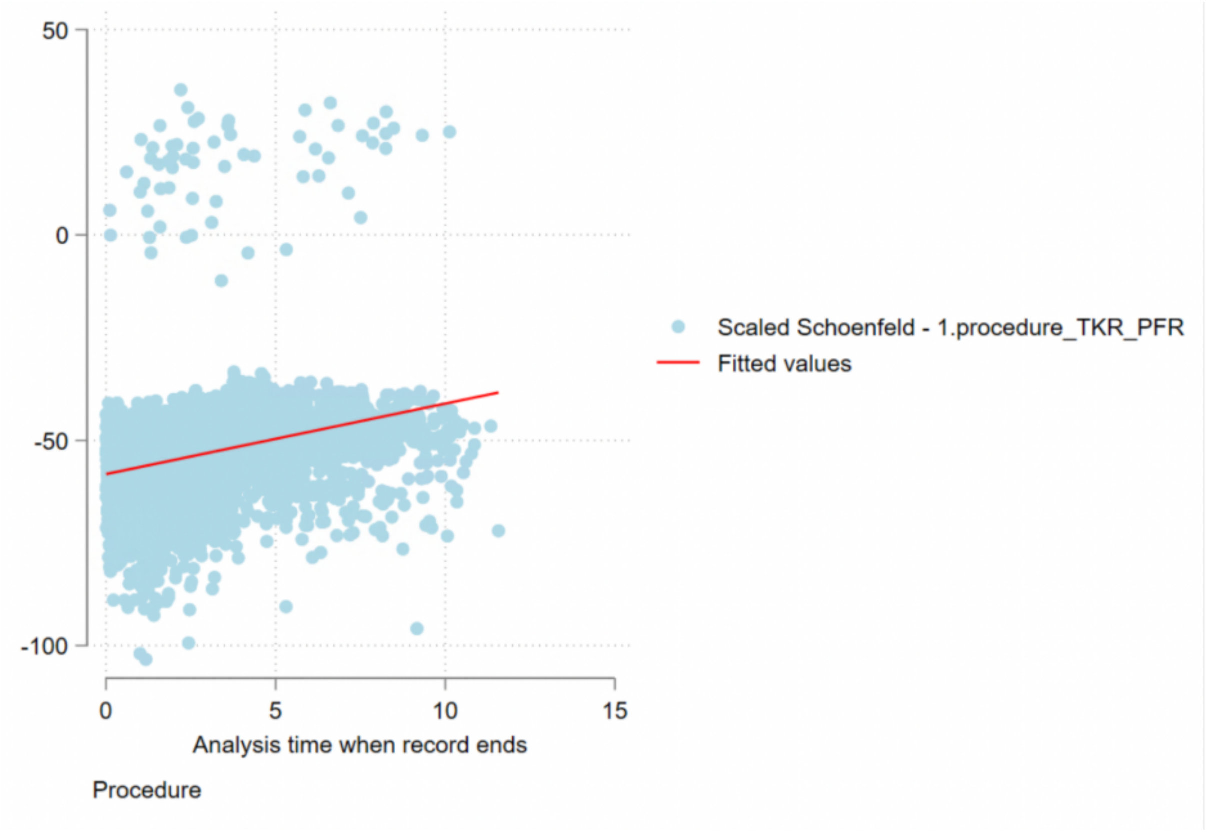

Figures 5b. Schoenfeld residuals for testing proportional hazards assumption for covariate type of procedure.

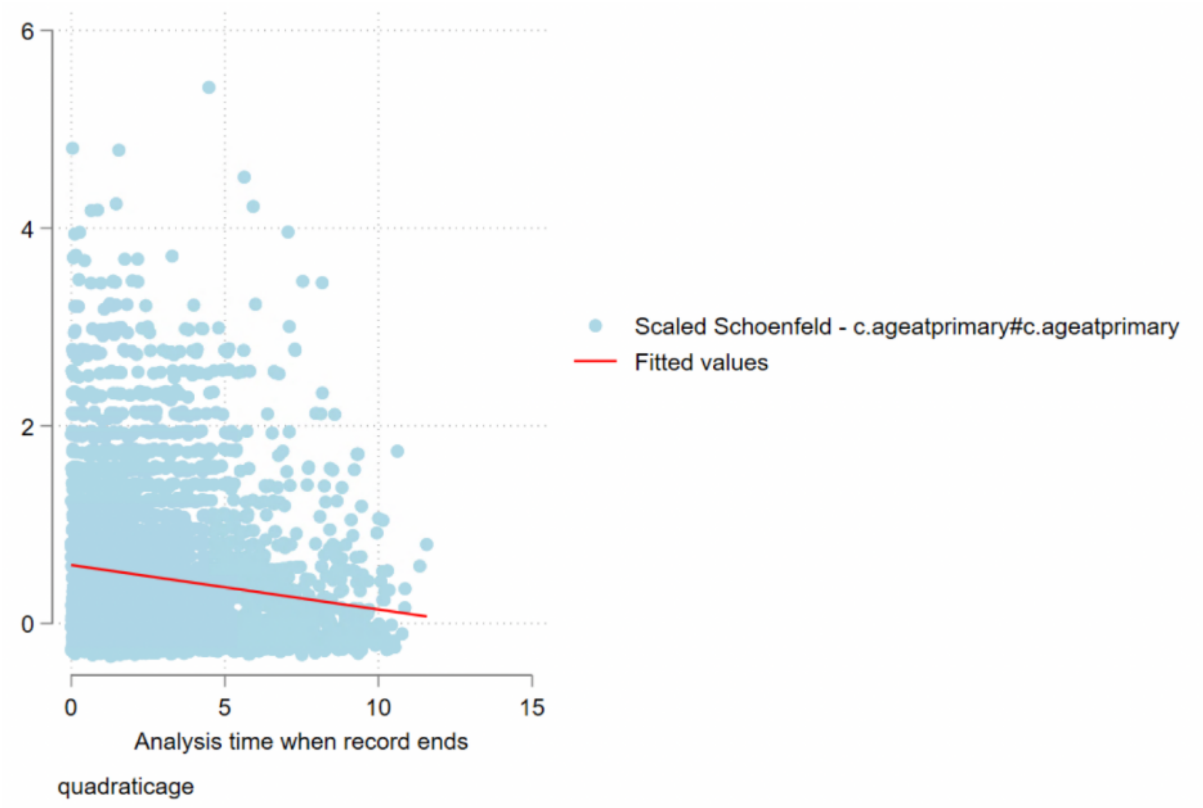

Figures 5c. Schoenfeld residuals for testing proportional hazards assumption for covariate age
